# Quantifying improvement of psychotic symptoms in clozapine-treated schizophrenia: clinical note analysis with large language models

**DOI:** 10.1038/s41598-026-39676-0

**Published:** 2026-02-13

**Authors:** Misa Matsumura, Keiichiro Nishida, Katsunori Toyoda, Kaori Kadoyama, Ryoichi Yano, Tetsufumi Kanazawa, Toshiaki Nakamura, Yosuke Morishima

**Affiliations:** 1 Education and Research Center for Clinical Pharmacy, Faculty of Pharmacy, Osaka Medical and Pharmaceutical University, Takatsuki, Osaka Japan; 2 Department of Neuropsychiatry, Faculty of Medicine, Osaka Medical and Pharmaceutical University, Takatsuki, Osaka Japan; 3 Translational research center, University Hospital of Psychiatry and Psychotherapy, University of Bern, Bolligenstrasse 111, Bern, 3000 Switzerland

**Keywords:** Psychosis, Natural language processing, Clinical notes, Language disturbance, Large language model, Brief psychiatric rating scale, Diseases, Health care, Medical research, Neuroscience, Psychology, Psychology

## Abstract

Symptoms of schizophrenia are often reflected in patients’ speech. Natural language processing (NLP) approaches enable quantitative assessment of language-related symptoms in schizophrenia. Previous applications have primarily focused on acute psychopathology or predicting the onset or relapse of psychosis rather than treatment-related improvements. Although electronic health records (EHRs) contain rich longitudinal data, unstructured notes hinder structured quantifications. We applied recent large language models (LLMs) to evaluate symptoms based on speech content recorded in EHRs. We analyzed 5,275 clinical notes from 30 patients with treatment-resistant schizophrenia undergoing clozapine treatment. Three state-of-the-art LLMs rated according to the Brief Psychiatric Rating Scale (BPRS). Complementary analysis included parts-of-speech (POS), bag-of-words (BoW), bigram and Linguistic Inquiry and Word Count (LIWC) analyses. LLM-based BPRS ratings revealed significant decreases in *Anxiety*, *Conceptual Disorganization*, *Suspiciousness*, *Unusual Thought Content*, *Hallucinatory behavior*, and *Depressive Mood* during clozapine treatment. POS analysis indicated an increased use of adjectives per sentence, while LIWC analysis revealed more positive emotional expressions during the later phase of treatment. These findings demonstrate that LLMs can extract clinically meaningful symptom information from unstructured clinical text and capture treatment-related changes in psychosis. This approach premises a low-burden method for supporting clinical judgment using routinely collected EHR data.

**Supplementary Information:**

The online version contains supplementary material available at 10.1038/s41598-026-39676-0.

## Introduction

 Schizophrenia is characterized by a broad range of symptoms, typically classified to positive, negative, and cognitive symptoms^1–3^. These symptoms often manifest in patients’ speech^4,5^. Positive symptoms, such as hallucinations and delusions, reflect a pathological excess or distortion of normal functioning, particularly in perception and thoughts. Negative symptoms, including affective flattening and avolition, involve reductions in fluent and voluntary speech and communication. Cognitive symptoms, which encompass impairments in higher-order cognitive functions (e.g., attention and problem-solving), can lead to difficulties in forming logical, complex thoughts and coherent communication.

Natural language processing (NLP), a computational approach to process natural language data, allows us to evaluate language features in a quantitative and reproducible way. Analyses of speech or written text from individuals with schizophrenia have revealed language disturbances in multiple linguistic domains^4–11^. These disturbances range from relatively simple characteristics—such as sentence length, part-of-speech tags, or speech rate—to more complex features, such as semantic coherence. Previous research focusing on simpler linguistic markers has reported decreased use of first-person pronouns, increased use of the third-person pronoun “they,” and frequent repetition of words^12,13^. These findings may be linked to delusional thinking and disruptions in self-reflection (see self-disorders)^14,15^. When examining semantic or discourse coherence, studies show that individuals with schizophrenia or those at clinical high risk for psychosis exhibit reduced semantic coherence^16–20^ which correlates with disorganized thought. Moreover, computational simulations have suggested that increased stochasticity and reduced memory span are associated with decreased semantic coherence^10^. However, other studies rather showed increased semantic coherence^11,21^. This increased semantic coherence was explained by compressed semantic space^22^. Accordingly, text-based analysis can reveal linguistic features that are closely related to the psychopathology of schizophrenia.

Formal assessments of speech and language in schizophrenia have typically been conducted through structured or semi-structured interviews, standardized neuropsychological tests, or tasks prompted by images or short films^6^. The responses to these assessments can be recorded as audio and then transcribed into text for further analysis. While most of these studies employed cross-sectional approaches, longitudinal approaches help identify clinically relevant language disturbances^23^. However, in routine clinical practice, time constraints often limit the feasibility of repeating formal measures for all patients. One potential way to overcome these constraints is by analyzing patients’ speech as recorded in electronic health records (EHRs).

EHRs contain various types of data, both structured and unstructured. Structured data include demographic information, vital signs, laboratory test results, medication histories, and billing codes for insurance providers—all of which can be standardized. In contrast, unstructured data consists of clinical notes and medical images. Although these unstructured data can provide valuable insights, analyzing them is typically time-consuming and complex, often requiring manual review^24^. Previous research involving EHR data in mental health settings has mainly focused on predicting diagnostic phenotypes or assessing risk of psychosis onset or hospitalization^25–28^. While such studies demonstrate the utility of EHRs for predicting psychosis onset or relapses, they provided less information about individual symptomatology.

In the current study, we leveraged recent advancements in large language models (LLMs) to evaluate complex linguistic features in clinical text^29,30^. As state-of-the-art models can process longer context and follow detailed instructions, we were able to design prompts that instructed LLMs to rate clinical notes derived from electronic health records (EHRs) according to the Brief Psychiatric Rating Scale (BPRS)^31,32^. To validate our approach^33–36^, we focused on patients with schizophrenia who received clozapine, a medication widely used for treatment-resistant cases that can improve both positive and negative symptoms^33^. In Japan, due to the risk of agranulocytosis associated with clozapine^36^, all patients receiving clozapine must be registered in a nationwide database and hospitalized when initiating treatment. Consequently, comprehensive clinical records covering the entire hospital stay are available for these patients over time. We extracted patients’ speech data transcribed by psychiatrists from clinical notes of EHRs and quantitatively analyzed the content by using LLMs. While patients’ speech transcribed by psychiatrists is relatively shorter and can be influenced by confirmatory biases, exploiting new technologies could open an opportunity to support clinical decision-making. Specifically, we designed prompts that instructed LLMs to assume the persona of a specialized psychologist and to rate each note according to the BPRS guidelines. To take into account heterogeneity among LLMs, we utilized three state-of-the-art LLMs. We hypothesized that improvements in schizophrenia symptoms during clozapine treatment would be reflected in observable changes in the linguistic features of patients’ recorded speech in clinical notes.

## Methods

### Participants

Inclusion criteria. We included patients diagnosed with treatment-resistant schizophrenia who began clozapine treatment at Osaka Medical and Pharmaceutical University Hospital between 2015 and March 31, 2023. In Japan, the prescription of clozapine treatment is regulated by guidelines: it is permissible only after the failure of two antipsychotics, and the patient must be hospitalized during initiation. Thus, all included patients are considered as treatment-resistant schizophrenia. Forty-five patients were identified from the Clozaril Patient Monitoring Service, a national registry for post-marketing surveillance.

Exclusion criteria. Patients were excluded if they (1) lacked a PANSS assessment or (2) had an initial PANSS assessment performed more than 30 days after clozapine initiation. Thirteen and two patients were excluded based on the first and second criteria, respectively. The remaining 30 patients were included in the current study. Written informed consent for the use of their clinical data for research purposes (general consent) was obtained from all participants. All procedures adhered to the principles of the Declaration of Helsinki, ethical guidelines for medical and biological research involving human subjects in Japan and Act on the Protection of Personal Information in Japan. The study protocol for using EHR data was approved by the Ethics Committee of Osaka Medical and Pharmaceutical University (Protocol ID; 2023-095-1).

### Extraction of speech from electronic clinical health records

After importing the EHRs of all included patients, we manually preprocessed the data.

Here are representative examples of subjective section of clinical notes:

**Example 1**

- Patient “Good morning.”
- Patient “I feel very tired and I couldn’t sleep well.”
- Patient “I also feel anxious.”

**Example 2**

- Patient “Good afternoon.”
- Patient “I still hear voice very often.”
- Patient “She (hallucinated voice) told me I am idiot.”
- Patient “Not any further problem.”

**Example 3**

- Psychiatrist “Good afternoon.”
- Patient “….”
- Psychiatrist “How do you feel today?”
- Patient “….”

First, from the subjective section of clinical notes, we extracted instances of patient’s speech transcribed by psychiatrists. After extraction, we excluded transcriptions corresponding to silence or muttering from the analysis. We then added periods to the end of each sentence that lacked punctuation to ensure accurate Japanese sentence splitting. In total, we obtained 22,716 sentences from 5,275 records.

### LLM analysis

LLM-based BPRS assessment was applied to patients’ speech recorded in clinical notes of EHRs. Due to the variability of outputs among LLMs, 3 recent models were included in our analysis: “gpt-oss-120b”^37^, “GLM-4.5-Air”^38^, and “Qwen3-Next-80B-A3B-Instruct”^39^. These three models were selected based on preliminary experiments using smaller models (approximately 30 billion parameters), which were unable to generate valid BPRS scores due to their relatively limited instruction-following capabilities. Thus, we have used the three models between 80–120B parameters. All models were obtained from Huggingface Hub (huggingface.com) and implemented using Python (version 3.12) and vLLM inference framework (version 0.11.0; https://github.com/vllm-project/vllm). Calculations were performed on UBELIX (https://www.id.unibe.ch/hpc), the HPC cluster at the University of Bern. We used 4 x NVIDIA H100 (96GB RAM) GPUs to run inference.

Each clinical record was subject to an LLM via a prompt designed to evaluate the 18 BPRS items. The LLM generated a score of each item between 0 and 1, instead of the conventional 1–7 scale. Because the prompt we used was considerably long (3623 characters in Japanese; 1084 words in the English prompt translated from the original Japanese one), the complete prompts are provided in the supplementary materials. Briefly the prompt was constructed as follows: The first part defined LLM’s persona in the system prompt as an expert in psychological assessment. Subsequent sections specified the general evaluation rule and definition of each BPRS item including guidelines on what to and what not to evaluate. Finally the prompt defined the JSON-style output format and included a few illustrative examples.

### Definition of baseline and treatment phase

To investigate changes in speech content during clozapine treatment, we defined a baseline period and three treatment phases. Day 1 was set as the first day of clozapine administration, and day N was defined as the day of hospital discharge. The pre-clozapine baseline (T0) period ranged from day − 30 to day 0. If a patient was admitted fewer than 30 days before clozapine initiation, outpatient records data was also included when it exists. We then divided the period from day 1 to day N into three equal parts: treatment phases 1, 2, and 3. On average, the baseline period lasted 14.2 days, and each treatment phase was 39.0 days. On average, initial PANSS assessment was conducted 8.4 days after clozapine initiation.

### Classical NLP analysis

To complement to LLM-based evaluation, we performed classical NLP analysis, including part-of-speech (POS) tags, Bag-of-Words (BoW), and bi-gram analyses. First, we split sentences in each record by using spaCy (https://spacy.io/) with its Japanese language model (ja_core_news_sm). Then, we used Janome (https://janome.mocobeta.dev/en/), which relies on the “mecab-ipadic-2.7.0–20070801.0 dictionary”, to assign POS tags for each word.

We then performed a BoW analysis focused on adjectives. The rationale to restrict the analysis to adjectives was based on our POS tag results, which indicated increase in adjectives over time. However, the amount of text from individual patients was insufficient for a patient-level BoW analysis, so for each treatment phase we aggregated data from all 30 patients. Using the POS tags from the previous step, we filtered out adjectives and converted each to its base form. Because certain variations in oral conversation imply similar meanings, we manually grouped synonyms into a single adjective shown in Table 4. The total number of sentences during the baseline period was significantly lower than in the treatment periods due to the shorter duration of the baseline. Hence, the direct comparison of raw BoW counts is not optimal. Instead, for each treatment period, we calculated the total number of sentences and scaled these values relative to the total sentences in the baseline period.

To investigate how adjective “no” was used, we computed bigrams containing “no”. In Japanese, “no” is used after another word to convey negation. Therefore, we focused specifically on “X – no” bigrams. For each sentence, we first excluded particles, auxiliary verbs and conjunctions, and then computed bigrams. Finally, we aggregated the bigram across all 30 patients.

### LIWC analysis

To evaluate the degree of emotional expression in patients’ speech, we used the Linguistic Inquiry and Word Count (LIWC) approach, a validated method to quantify psychologically meaningful words^40,41^. For each patient and treatment phase, we preprocessed the speech text as described above to divide into words. Then, we computed the word counts of “affect”, “positive emotion”, and “negative emotion” with the Japanese version of the LIWC dictionary 2015^42^, and scaled by a total word count of the treatment phase for each patient. We computed LIWC features with in-house python scripts.

### Factor score of clinical rating scales

Lastly, we examined the associations between To examine the association of conventional NLP measures including LIWC with human-rated PANSS score and LLM-based BPRS, we categorized items based on meta-analysis of factor analysis on PANSS and BPRS^43,44^. Each of PANSS factors include the following items. Affect includes G2 Anxiety, G6 Depression, G3 Guilt, G4 Tension, G1 Somatic concern. Positive symptom includes, P1 Delusions, G9 Unusual thought content, P3 Hallucinatory behavior, P6 Suspiciousness and persecution, P5 Grandiosity. Negative symptom includes N2 Emotional withdrawal, N1 Blunted affect, N4 Passive apathetic social withdrawal, N6 Lack of spontaneity, N3 Poor rapport, G7 Motor retardation, and G16 Active social avoidance. Disorganization includes P2 Conceptual disorganization, G11 Poor attention, N5 Difficulty in abstract thinking, G13 Disturbance of volition, N7 Stereotyped thinking, G5 Mannerisms and posturing, and G15 Preoccupation. Resistance includes P7 Hostility, G14 Poor impulse control, P4 Excitement, G8 Uncooperativeness. For BPRS factor analysis, Mannerism and posturing, Tension, and Motor retardation were excluded, because those items were solely evaluated by observed behavior. Thus, each BPRS factor includes the following items. Affect includes Depression, Guilt, Anxiety, and Somatic Concern. Positive symptom includes Unusual Thought Content, Hallucinations, Suspiciousness, Grandiosity, and Disorganization. Activation includes Excitement, and Hostility. Negative symptom includes Blunted Affect, Emotional Withdrawal, Uncooperativeness, and Disorientation. To compute factor scores, we simply summed up item score for each factor, and the scores were used for the association analysis.

### Statistics

We performed repeated one-way ANOVA tests to determine whether the number of records, the number of sentences, and sentence length changed over the course of treatment. Post-hoc comparisons were then conducted to compare each treatment phase with the baseline period. Because sentence length (words per sentence) differed significantly among the treatment phases, we used a linear mixed-effects model to test whether the number of adjectives, adverbs, nouns, or verbs changed over time. In this model, treatment phase and sentence length were included as fixed effects, and subject was included as a random effect. The main effect of treatment phase was evaluated using analysis of variance within the mixed-effects model, followed by post-hoc comparisons comparing each treatment phase with the baseline period. p-values for these post-hoc comparisons were adjusted by Dunnett’s correction for multiple comparisons. A p-value of less than 0.05 was considered significant. All statistical analyses were performed in R (https://cran.r-project.org/) using the “lme4” and “lmerTest” packages. We used “fmsb” and “ggplot2” for data visualization.

## Results

Patients’ characteristics and demographics are shown in Table 1. The mean human-rated PANSS score at the start of clozapine treatment was 98.5, indicating that symptoms were considerably severe (Table 1). During the baseline period, the numbers of records and sentences were significantly lower than in any of the treatment phases (Recorded data, F(3,87) = 20.34, *p* < 0.001; Included sentences F(3,87) = 11.52, *p* < 0.001) (Table 2. This was due to a shorter baseline period (14.2 days) compared to each treatment phase (39.0 days). However, the length of sentences at the baseline was significantly longer than in the treatment phases (F(3,87) = 6.595, *p* < 0.001) (Table 2).

**Table 1:** Patients’ characteristics and demographics.

Measure	Patients (*n* = 30)	
Age, *M (SD)*	42.0	(12.5)
Sex, *N*		
Male	8	(26.7%)
Female	22	(73.3%)
Patients who continued treatment with CLOZ until the day of discharge, *N*	22	(73.3%)
CLOZ maintenance dose, *M (SD)*	310.8	(187.0)
Patients whose clozapine therapy was discontinued, *N*	8	(26.7%)
Clozapine dose at discontinuation, *M (SD)*	131.3	(66.5)
Average of first PANSS total score, *M (SD)*	98.5	(28.1)
Mean difference in the number of days between the date of the first PANSS measurement and the date of clozapine initiation, *M (SD)*	8.3	(16.5)

M = mean, SD = standard deviation.

**Table 2:** Descriptive statistics of text data used by treatment phase.

	Baseline Mean (SD)	Phase 1 Mean (SD)	Phase 2 Mean (SD)	Phase 3 Mean (SD)	RM ANOVA F, *p*-value
Number of records					
	25.333	56.1***	50.9***	43.4**	F(3,87) = 20.34
	(19.295)	(25.7)	(23.9)	(21.4)	*p* < 0.001
Number of sentences					
	124.200	231.1***	218.3**	183.5	F(3,87) = 11.52
	(95.930)	(110.2)	(120.5)	(95.1)	*p* < 0.001
Average number of words per sentences					
	9.393	8.067***	8.375	8.457	F(3,87) = 6.59
	(2.576)	(2.303)	(2.602)	(2.484)	*p* < 0.001

**Fig. 1:**
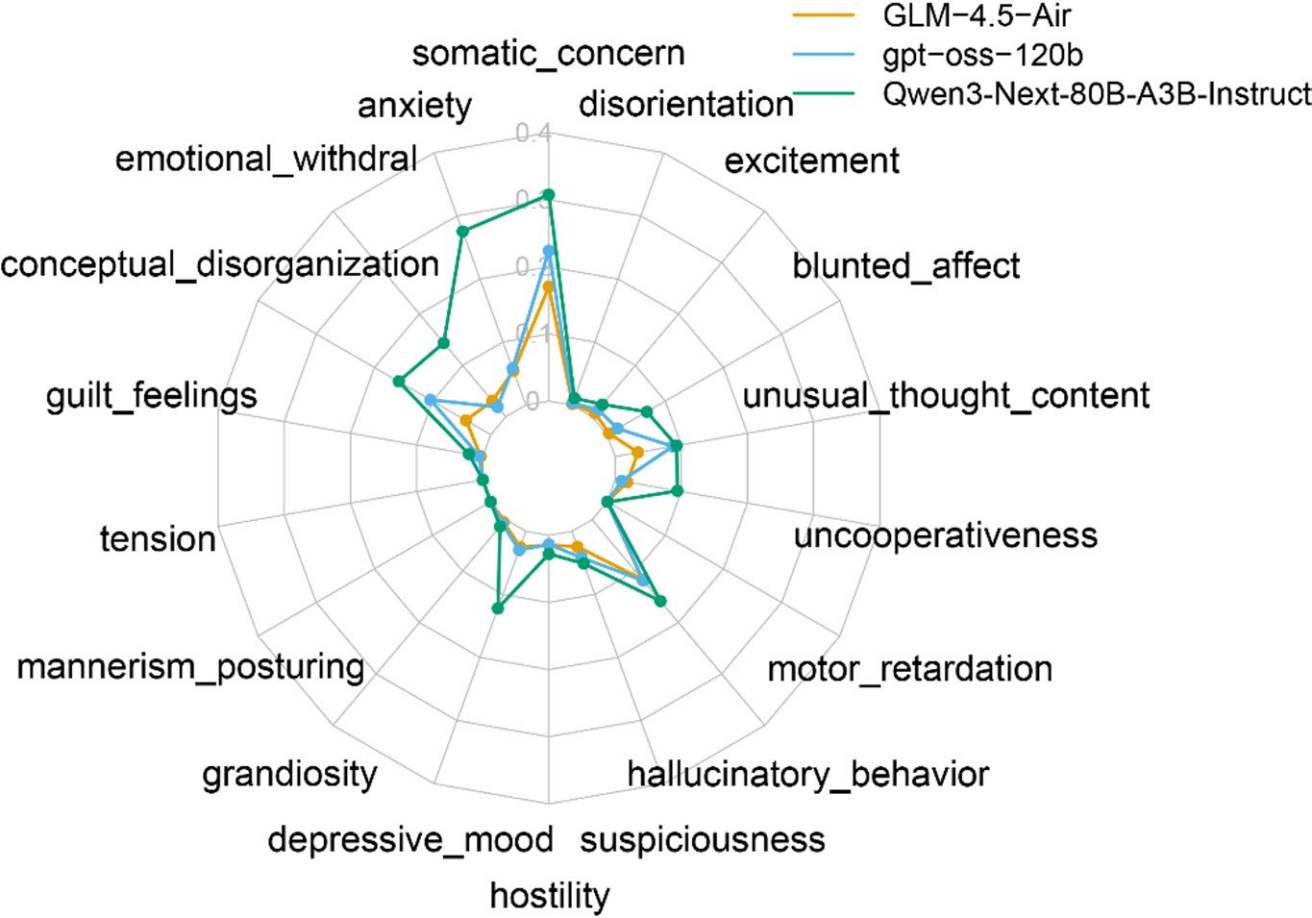
Grand average of LLM-based BPRS rating. An overall pattern of 18 items are consistent across the three LLMs. Items rated solely based on behavioral observations (*tension*, *mannerism* and *posturing*, and *motor* *retardation*) were rated almost zero. One model (Qwen3, green) tends to rate higher score.

Next to assess symptoms using LLMs, we computed LLM-based BPRS scores. However, as no prior validation exists for the ability of LLMs to evaluate speech data according to BPRS. Thus, we first conducted a validation analysis of the three models we have employed. In BPRS, some items need to be evaluated based on subjective reports, while others are evaluated based on observable behavior and speech^8^. In particular, *Tension*,* Mannerisms and Posturing*, and *Motor Retardation* must be evaluated solely based on observed behavior. Therefore, these items cannot be appropriately assessed by LLMs. Figure 1 shows the grand average scores of 5275 records for each BPRS item. All three models produced relatively low scores in these three items, and among these three models, “gpt-oss-120b” and “GLM-4.5-Air” showed consistent performance. For remaining 15 items, we have calculated the correlation between human-rated PANSS and LLM-based BPRS scores for each item during the baseline period. We found significant positive correlations of *Hallucinations*,* Disorganization*, and *Uncooperativeness* for all three LLMs, and significant positive correlation of *Excitement* and *Hostility* for two LLMs (GLM-4.5-Air and gpt-oss-120b) (Supplementary Table 4).

**Fig. 2:**
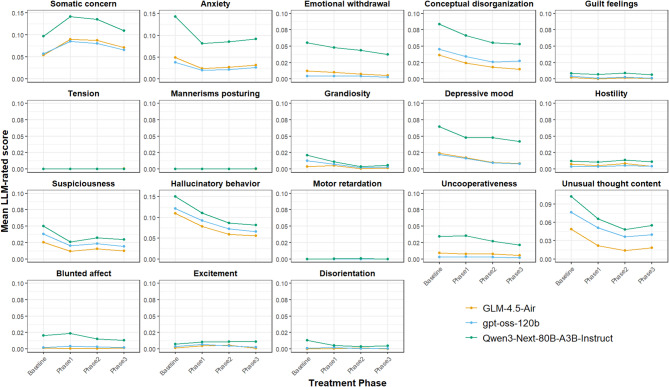
LLM-based BPRS rating during treatment. Mean values of LLM-based BPRS rating across participants were plotted during the course of treatment. All three models showed similar pattern during the course of treatment. One model (Qwen3, green) showed higher scores in some items.

We then examined whether LLM-based assessment could identify the changes in symptoms during clozapine treatment. ANOVA analysis revealed all three LLMs consistently rated significant changes in *Somatic Concerns* (*p* < 0.05), *Anxiety* (*p* < 0.01), *Conceptual Disorganization* (*p* < 0.01), *Depressive Mood* (*p* < 0.05), *Suspiciousness* (*p* < 0.05), *Hallucinatory Behavior* (*p* < 0.001), *and Unusual Thought Content* (*p* < 0.001) during treatment (Fig. 2, Supplementary Tables 1–3). Among these 7 items, all but *Somatic Concerns* significant decrease compared to the baseline, whereas *Somatic Concerns* initially increased after starting clozapine administration and then decreased over time.

While LLM outputs provide holistic summary statistics of the text, the linguistic characteristics of speech contents remain elusive. To complement LLM-based analysis, we conducted conventional NLP approaches including POS tagging, BoW, bi-gram, and dictionary-based analysis (LIWC). We calculated the average number of POS tags per sentence. Because sentence lengths differed significantly between baseline and treatment phases (Table 2), we used a linear mixed-effects model to adjust for this potential influence. As shown in Table 3, we found that the number of adjectives per sentence (F(3,95.80) = 8.079, *p* < 0.001) and number of verbs per sentence (F(3, 96.23) = 2.975, *p* = 0.035) changed significantly. However, number of adverbs (F(3,96.75) = 2.186, *p* = 0.094) and noun (F(3,93.34) = 0.0822, *p* = 0.96) did not exhibit significant change. Post-hoc analysis revealed that numbers of adjectives per sentence were significantly increased from the baseline (Phase 1, *p* < 0.001; Phase 2, *p* < 0.001; Phase 3, *p* = 0.0021) and that the number of verbs per sentence only in Phase 3 was significantly decreased from baseline (*p* = 0.013).

**Table 3:** Part of speech tag count per sentence.

POS	Baseline Mean (SD)	Phase 1 Mean (SD)	Phase 2 Mean (SD)	Phase 3 Mean (SD)	Time main effect F, *p*-value
adjective					
	0.214	0.275***	0.282***	0.266**	F(3, 95.80) = 8.08
	(0.075)	(0.082)	(0.101)	(0.080)	*p* < 0.001
adverb					
	0.226	0.235	0.224	0.239	F(3, 96.75) = 2.19
	(0.099)	(0.117)	(0.096)	(0.096)	*p* = 0.094
Noun					
	2.221	1.829	1.908	1.929	F(3, 93.34) = 0.08
	(0.739)	(0.642)	(0.677)	(0.748)	*p* = 0.96
Verb					
	1.496	1.188	1.236	1.219*	F(3, 96.23) = 2.98
	(0.552)	(0.484)	(0.526)	(0.502)	*p* = 0.035

Dunnett’s correction for multiple comparison was applied to post-hoc comparison to baseline. SD = standard deviation. * *p* = 0.013; ** *p* = 0.0021, *** *p* < 0.001.

Because the POS tagging analysis indicated that the increase in adjectives might reflect a treatment effect, we performed a Bag-of-Words (BoW) analysis by counting the occurrences of each adjective in the patients’ speech. The ten most frequent adjectives are shown in Table 4. As the number of sentences varied between the baseline and treatment phases, we normalized the adjective counts by the number of sentences in each phase so that changes could be compared with baseline values. The BoW analysis revealed an increase in adjectives such as “good”, “pleasant”, “tired,” “sleepy,” “terrible” and “scary” and a decrease in the use of “no” and “bad” over the course of clozapine treatment.

Among adjectives listed in Table 4, all but “no” covey clear meanings, while “no” needs further context to understand how it was used. To clarify this ambiguity, we further performed bigram analysis. In Japanese language, an adjective of “no” is usually preceded by other words in a negation phrase. Thus, we counted numbers of “X-no” bigrams (Table 5). The bigram analysis revealed that “no” is used in context of unresponsive answers, such as “nothing”, “nothing in particular”, and “no change”. Thus, these results suggest that decrease in “no” reflects reduction of detached conversations.

**Table 4:** Top 10 adjectives appeared in BoW analysis.

Rank		Number of occurrences					Values scaled by total number of sentences by phase ^a^			
	adjective	Baseline	Phase1	Phase2	Phase3	Total	Phase1	Phase2	Phase3	Baseline vs. Phase3
										**(% change)**
1	good	157	329	379	342	1207	176.8	215.6	231.4	47.4
2	tired	109	320	330	225	984	172	187.7	152.2	39.7
3	no	161	342	231	165	899	183.8	131.4	111.7	−30.6
4	sleepy	16	242	142	99	499	130	80.8	67	318.8
5	bad	53	84	80	73	290	45.1	45.5	49.4	−6.8
6	painful	35	67	56	52	210	36	31.9	35.2	0.6
7	willing	26	40	41	37	144	21.5	23.3	25	−3.7
8	terrible	12	30	37	22	101	16.1	21	14.9	24.1
9	scary	10	23	32	32	97	12.4	18.2	21.7	116.6
10	pleasant	6	10	32	44	92	5.4	18.2	29.8	396.3

^a^ Number of sentences in each treatment phase divided by number of sentences in baseline.

**Table 5:** Top 6 “X - no” bigram.

Ranking	bigram meaning	counts
1	Nothing	57
2	Nothing in particular	36
3	No change	28
4	Not much	17
5	No way	13
6	Hardly	10

Only no less than 10 counts of “X - no” bigrams are listed.

Our analyses of adjectives suggest increased emotional expression during the clozapine treatment. To further address changes in emotional expression, we performed LIWC analysis to compute word counts related to emotional expression. We found that a significant main effect of treatment phase in the “positive emotion” category (F(3,87) = 4.151, *p* = 0.008), and post-hoc comparison revealed significant increase in positive emotional words in the treatment phase 3 (*p* = 0.0027) (Table 6). In contrast, there was no significant main effect in affect and negative emotion categories (affect: F(3,87) = 2.23, *p* = 0.09; negative emotion: F(3,87) = 0.04, *p* = 0.98).

**Table 6:** LIWC analysis.

Emotion	Baseline Mean(%) (SD)	Phase 1 Mean(%) (SD)	Phase 2 Mean(%) (SD)	Phase 3 Mean(%) (SD)	Time main effect F, *p*-value
Affect					
	4.149	4.560	4.476	4.972	F(3,87) = 2.23
	(0.019)	(0.020)	(0.016)	(0.022)	*p* = 0.09
Positive					
	2.075	2.336	2.354	2.878**	F(3,87) = 4.151
	(0.012)	(0.018)	(0.014)	(0.020)	*p* = 0.008
Negative					
	1.765	1.769	1.713	1.731	F(3,87) = 0.04
	(0.009)	(0.008)	(0.009)	(0.011)	*p* = 0.98

Dunnett’s correction for multiple comparison was applied to post-hoc comparison to baseline. SD = standard deviation. ** *p* = 0.0027.

We further examined whether conventional NLP metrics at baseline were associated with the human-rated baseline PANSS scores. The Disorganized Orientation and Resistance factors were significantly correlated with sentence length and with the number of nouns and verbs per sentence (*p* < 0.05; Supplementary Table 5). None of the human-rated PANSS factors was significantly correlated with the LIWC metrics (all *p* > 0.05).

Lastly, we examined the associations between LLM-based BPRS score and conventional NLP measures to fill the gap between these two approaches. We found that BPRS Affect, Positive and Negative factors were significantly correlated with sentence length, number of nouns and verbs per sentence (*p* < 0.05) for all three LLMs. BPRS Affect, Positive, and Activation factors were significantly correlated with number of adjectives per sentence (*p* < 0.05). BPRS Affect and Activation factors were significantly correlated with number of adverbs per sentence. We also found significant negative correlations between BPRS affect and LIWC positive words, and positive correlations between BPRS Affect and LIWC negative words and between BPRS activation and LIWC positive words (Supplementary Table 6).

## Discussion

In the current study, we leveraged the capabilities of LLMs to rate symptoms based on speech content recorded in clinical notes of EHRs. We further applied conventional NLP methods to characterize changes in linguistic features and to aid interpretation of the LLM-based ratings. Our findings are summarized in four main points. First, LLM-based BPRS rating revealed significant decrease in *Anxiety*,* Conceptual Disorganization*,* Suspiciousness*,* Unusual Thought Content*, and *Depressive Mood* during treatment, and increase in *Somatic Concerns* when initiating clozapine. Second, the POS analysis revealed an increased use of adjectives per sentence over the course of treatment. Third, we observed increased expressions of emotional states and physical conditions, while decreased expression of “no changes or notable concerns”. Lastly, LIWC analysis revealed increased positive emotion in the last third of a treatment period.

The current study focused on text data derived from EHRs, requiring no additional burden to patients. By combining various NLP methods, including LLM-based BPRS rating, we characterized how psychotic symptoms were improved in patients with treatment-resistant schizophrenia.

Previous studies on language characteristics in schizophrenia and psychotic spectrum disorders have primarily analyzed structured speech data collected outside routine clinical practice. These studies typically examined linguistic markers such as POS tags, the use of adjectives and emotional adjectives (often using LIWC), and semantic coherence^7,12,16,18–20,45^. Consistent findings across these studies include reduced use of adjectives, adverbs, verbs, and first-person pronouns, as well as decreased syntactic complexity and semantic coherence. Our finding of increasing adjectives during the treatment is line with previous research.

Applications of large language models in psychiatric research have grown rapidly in recent years^27,46,47^. LLMs have been used to predict the onset of first episode psychosis^25^ and hospitalization risks^48^. In this study, we employed state-of-the-art LLMs to evaluate symptoms according to BPRS, a widely used clinical assessment scale. As a proof of concept, we validated our approach using several BPRS items that are rated solely based on behavioral observation and comparison with human-rated PANSS score at the baseline period^8^. Indeed, “*Tension*”, “*Mannerisms* and *Posturing*”, and “*Motor* *Retardation*” were consistently rated near zero across the three models, supporting their use as “negative” sanity-check items. Furthermore, five items showed significant positive correlations between human-rated PANSS and LLM-based BPRS (Supplementary Table 4). One model (Qwen3-Next-80B) produced consistently higher scores across most items and demonstrated less items of significant item-wise correlation with human-rated PANSS score. Furthermore, LLM-based BPRS scores were generally low even during the baseline period (Fig. 2). We attribute this to a key limitation of clinical notes: the recorded text is brief—approximately four sentences per record (Table 2)—and therefore may not capture all symptoms present at a given time point. Indeed, heatmaps of the LLM-based BPRS scores suggest that some items are expressed in one record, whereas other items may be expressed in another (Supplementary Fig. 1). Despite such heterogeneity in absolute scores, all three LLMs consistently rated significant changes when observed, indicating that internal consistency is confirmed across the three LLMs. We further observed decrease in *Conceptual Disorganization*,* Suspiciousness*,* Unusual Thought Content*, indicating that LLMs were able to capture the clinically relevant symptomatic changes through clinical notes. We also observed an increase in *Somatic Concern* at the beginning of clozapine treatment, likely reflecting the emergence of side effects such as fatigue and sleepiness demonstrated by the BoW analysis. Indeed, sleepiness, tachycardia, hypotension and constipation are known as common adverse effects of clozapine^33,35,36^. While sleepiness was detected by the BoW analysis, other common adverse effects were also reflected in *Somatic Concern* of LLM-based BPRS rating. These results demonstrate the potential utility of LLMs for large-scale symptom assessment from clinical notes.

Concerning emotional expression, the LLMs identified decreases in *Anxiety* and *Depressed Mood*, but no significant change in *Emotional Withdrawal* or *Blunted Affect* during treatment. LIWC analysis revealed an increase in positive emotional words, but no change in negative emotional words. The correlation analysis between LLM-based BPRS factor scores and conventional NLP metrics revealed that BPRS Affect factor, which includes *Anxiety* and *Depressed Mood*, was correlated with all conventional NLP metrics except overall affect words of LIWC (Supplementary Table 6). In contrast, *Emotional Withdrawal* and *Blunted Affect* represent internalized symptoms that are less likely to be explicitly reflected in text and thus remain difficult for LLMs to evaluate. These results suggest that LLM-based BPRS and conventional NLP measures were complementary in capturing emotional expression reflected in transcribed speech in clinical notes.

While conventional methods like LIWC are reproducible, such methods cannot capture the complex, context-dependent nuances of human language. In contrast, our study exploited the capabilities of LLMs, revealing a significant improvement in various symptoms during clozapine treatment. The correlation analysis of these two approaches revealed that they are not independent nor orthogonal, but rather complementary to each other. POS tag metrics were positively correlated with BPRS Affect and Positive factors, while negatively correlated with Activation and Negative factors (Supplementary Table 6). However, conventional NLP measures had very weak association with PANSS factors (Supplementary Table 5). Therefore, their relevance to symptomatology is limited, compared to LLM-based approaches. These results suggest that even general-purpose LLMs can detect meaningful clinical change and add further interpretation of changes observed by conventional NLP approaches.

Descriptive statistics of the source text data revealed longer sentence length in the baseline pre-clozapine period, compared to clozapine treatment period. We consider this was due to the nature of processes during hospitalized treatment. At the admission, various types of open-ended questions are asked, while questions asked towards the discharge are relatively limited. A significant increase in adjectives we observed is consistent with previous studies, while a significant decrease in verb counts per sentence, showing the opposite direction (Table 3). We believe this discrepancy may be derived from the difference in source data. Most of previous studies utilized semi-structured interviews or a story-telling elicited by a picture or key word, and researchers made a substantial effort to standardize these data. While such an approach yields a relatively long and standardized text, more suitable for text analysis, our data was extracted directly from EHRs, where typically intensive assessment made at admission and more limited assessment when clinical conditions are relatively stable or close to discharge.

There are potential biases remaining in clinical notes. First, clinicians may have a confirmatory bias on the effectiveness of treatment, therefore symptoms recorded in clinical notes may be underrated. An independent and blind human rating can detect this bias, however this is not available in the current study. Second, recorded speech of patients is subject to the bias of the recording individual (in this case, a psychiatrist), and there may be considerable variabilities among different clinicians^49^.

The BoW analysis of adjectives revealed changes in four clusters (Table 4). The first cluster included positive emotional adjectives such as “good” and “pleasant”, reflecting increased expression of positive feelings. The second cluster included negative emotional adjectives, such as “terrible” and “scary”, and the third cluster included adjectives related to sedation, such as “tired” and “sleepy”. The last cluster showed a decrease in expression of “no”. While the polarity of emotion in the first cluster seems opposite from the second and third clusters, overall findings suggest that the patients exhibited more emotional expression regardless of positivity or negativity. We additionally performed “X-no” bigram analysis to interpret the decrease in “no”. Examination of bigrams revealed that “no” was often used in phrases such as “no change” or “Nothing in particular” (Table 5), typically in response to the questions about the current symptoms or feelings asked by a doctor. Thus, fewer use of “no” might indicate that patients responded more proactively rather than giving fixed, and disengaged answers, implying an improvement in avolition. In sum, these changes in adjective use suggest that patients’ emotional expression and motivation improved, as revealed by their speech content in EHRs.

The LIWC analysis revealed an increase in positive emotional words in the final third of the treatment period. LIWC is a validated approach to quantify psychologically meaningful words^40,41^, and previous studies have revealed reduced positive emotional expression in patients with chronic schizophrenia^50–52^. In line with these previous studies, we found an increase in positive emotional expression during the clozapine treatment. It is important to note that the LIWC and BoW of adjectives analyses assess different sets of words. While our BoW analysis only focuses on adjectives, LIWC covers adjectives as well as nouns and verbs. A major limitation of the LIWC approach is its reliance on a predefined dictionary of psychologically relevant terms, which makes it difficult to capture disengaged responses, such as “nothing in particular”, identified through bigram analysis. These complementary results are consistent with decrease in LLM-based rating of *Depressed Mood*.

In the current study, our analysis of clinical notes in EHRs revealed an improvement and increase in emotional expression over the course of treatment. Emotional expression is a core domain of negative symptoms, and its improvement is highly associated with better psychosocial functioning in patients with schizophrenia and clinical high risk for psychosis^51,53,54^. Reduced emotional expression could hinder maintaining interpersonal relationships, leading to social isolation and a lower quality of life. Indeed, improvement of emotional expression has been shown to enhance the quality of life not only for patients but also for caregivers^55^. Indeed, a previous study successfully extracted patients’ emotion and mood from clinical notes data in EHRs^56^. Therefore, evaluating emotional expression in EHRs may provide a new way to investigate the psychosocial aspects of psychiatric patients.

The current study employed a longitudinal design, using patients’ speech as recorded in EHRs to reveal their emotional states during treatment. Our findings shed light on patients’ emotional changes over time. Most of previous research on speech text analysis in schizophrenia spectrum disorders utilized cross-sectional data^12,17,19,23,45,57,58^, which provided detailed insight into language disturbances, but could not address how these disturbances evolve during the treatment. Previous longitudinal studies have focused on predicting psychosis onset or hospitalization rather than analyzing psychosocial aspects^23^.

There are advantages and disadvantages to using EHR data. Using EHRs offers three main advantages. First, using existing records does not incur any additional burden on patients. Second, longitudinal data can be analyzed without significant extra effort. Third, clinical notes recorded in EHRs are documented by experts and tend to use standardized terminology. However, there are also drawbacks. First, the recorded text is neither fully structured nor standardized, resulting in substantial heterogeneity among different recorders. Second, recorded patients’ speech might be rephrased, because it relies on the memory of clinicians due to time lag between an interview and documentation. Third, the richness of documentation varies according to the treatment process (e.g. more intensive at admission and less intensive later). Overall, future research using EHRs should exploit the advantages and address these drawbacks. Additionally, we have manually extracted patients’ speech from the clinical notes. This limits the speed of data processing. We expect that large language models will allow automatic extraction of patients’ speech in future research.

This study has several limitations. First, speech content does not represent a direct transcription of patient speech, rather it is filtered through psychiatrists. Thus, content recorded by psychiatrists are presumably biased by therapists’ thoughts. In particular, this could be exaggerated towards discharge due to confirmation bias. Additionally, different clinicians may record the same information in varying ways, introducing further variations and biases. Including records by nurses may help to mitigate such biases. Second, formal assessment of symptom severity was available only at the time of admission. This made it difficult to address the association between symptoms and NLP features including LLM-based BPRS ratings and conventional features. Third, patients’ speech transcribed in clinical notes tends to be shorter than semi-structured interviews, and therefore pooling a certain period is crucial, instead of an observation on a single day. Fourth, the included number of patients was relatively small. Lastly, muttering or silence were not consistently recorded and they were excluded from conventional NLP analysis, while those expressions convey emotional expressions^59^.

## Conclusion

Our findings demonstrate that LLMs can detect the improvement of symptoms from patients’ speech transcribed by clinicians in EHRs during treatment. The results are supported by conventional characterizations of linguistic features. This approach may prove useful in future evaluation of psychosocial functioning in psychiatric patients during the treatment processes.

## Supplementary Information

Below is the link to the electronic supplementary material.

Supplementary Material 1

## Data Availability

EHR data is available only after the approval of the local ethics committee due to the privacy protection of the Act on the Protection of Personal Information in Japan. To request the access to the data, please contact to the corresponding author ([yosuke.morishima@unibe.ch](mailto: yosuke.morishima@unibe.ch)). Upon publication, the code used for the LLM-based BPRS rating will be made available at: [https://github.com/ymorishi/bprs\_ja](https:/github.com/ymorishi/bprs_ja).
