# Supplementary material for: Quantifying improvement of psychotic symptoms in clozapine-treated schizophrenia: clinical note analysis with large language models
Source: Sci Rep. 2026 Feb 13;16:8835. doi: 10.1038/s41598-026-39676-0 (PMC12982610; doi:10.1038/s41598-026-39676-0)
Supplement: Supplementary file 1 — Supplementary Material 1 [file 41598_2026_39676_MOESM1_ESM.docx]

**Supplementary materials for**

**Quantifying improvement of psychotic symptoms in clozapine-treated schizophrenia: Clinical note analysis with large language models**

Misa Matsumura^1^, Keiichiro Nishida^2^, Katsunori Toyoda ^2^, Kaori Kadoyama^1^, Ryoichi Yano^1^, Tetsufumi Kanazawa ^2^, Toshiaki Nakamura^1^, Yosuke Morishima^3^*

1. Education and Research Center for Clinical Pharmacy, Faculty of Pharmacy, Osaka Medical and Pharmaceutical University, Osaka, Japan

2. Department of Neuropsychiatry, Faculty of Medicine, Osaka Medical and Pharmaceutical University, Osaka, Japan

3. Translational research center, University Hospital of Psychiatry and Psychotherapy, University of Bern, Bern, Switzerland

Corresponding author

Yosuke Morishima

Translational research center, University Hospital of Psychiatry and psychotherapy,

University of Bern, Bern, Switzerland

yosuke.morishima@unibe.ch

This file includes

- Supplementary Tables 1-6
- Supplementary Figure 1
- BPRS evaluation prompt in Japanese
- English translated BPRS evaluation prompt

Supplementary Table 1

Descriptive statistics and ANOVA table of BPRS rating made by “GLM-4.5-Air”

|  | **Baseline** | **Phase 1** | **Phase 2** | **Phase 3** | **ANOVA** |
| --- | --- | --- | --- | --- | --- |
|  | **Mean (SD)** | **Mean (SD)** | **Mean (SD)** | **Mean (SD)** | **F, p-value** |
|  |  |  |  |  |  |
|  |  |  |  |  |  |
| **somatic_concern** | 0.055 | 0.090** | 0.088* | 0.072 | 4.059, 0.01* |
|  | (0.059) | (0.075) | (0.082) | (0.082) |  |
| **anxiety** | 0.050 | 0.024* | 0.027* | 0.032 | 4.356, 0.007** |
|  | (0.047) | (0.021) | (0.03) | (0.037) |  |
| **emotional_withdrawal** | 0.013 | 0.010 | 0.008 | 0.006 | 1.072, 0.365 |
|  | (0.023) | (0.015) | (0.015) | (0.008) |  |
| **conceptual_disorganization** | 0.037 | 0.024 | 0.018 | 0.015* | 4.71, 0.004** |
|  | (0.055) | (0.040) | (0.025) | (0.029) |  |
| **guilt_feelings** | 0.002 | <0.001 | 0.002 | <0.001 | 1.101, 0.353 |
|  | (0.006) | (0.001) | (0.006) | (0.003) |  |
| **tension** | 0 | 0 | 0 | <0.001 | - |
|  | (0) | (0) | (0) | (0.003) |  |
| **mannerisms_posturing** | 0 | 0 | 0 | 0 | - |
|  | (0) | (0) | (0) | (0) |  |
| **grandiosity** | 0.004 | 0.005 | <0.001 | 0.002 | 1.657, 0.183 |
|  | (0.008) | (0.018) | (0.003) | (0.009) |  |
| **depressive_mood** | 0.024 | 0.017 | 0.010* | 0.008 | 3.649, 0.016* |
|  | (0.04) | (0.021) | (0.018) | (0.011) |  |
| **hostility** | 0.007 | 0.005 | 0.008 | 0.004 | 0.71, 0.549 |
|  | (0.016) | (0.011) | (0.017) | (0.009) |  |
| **suspiciousness** | 0.025 | 0.012 | 0.016 | 0.013 | 3.157, 0.029* |
|  | (0.036) | (0.020) | (0.023) | (0.021) |  |
| **hallucinatory_behavior** | 0.110 | 0.078* | 0.059** | 0.056* | 7.83, <0.001*** |
|  | (0.124) | (0.082) | (0.073) | (0.069) |  |
| **motor_retardation** | 0 | 0 | 0 | 0 | - |
|  | (0) | (0) | (0) | (0) |  |
| **uncooperativeness** | 0.009 | 0.008 | 0.008 | 0.005 | 0.278, 0.841 |
|  | (0.023) | (0.018) | (0.021) | (0.015) |  |
| **unusual_thought_content** | 0.049 | 0.022* | 0.014** | 0.018* | 9.37, <0.001*** |
|  | (0.064) | (0.036) | (0.026) | (0.030) |  |
| **blunted_affect** | 0.002 | <0.001 | <0.001 | 0.002 | 0.756, 0.522 |
|  | (0.005) | (0.002) | (0.001) | (0.004) |  |
| **excitement** | 0.002 | 0.005 | 0.006 | 0.001 | 3.541, 0.018* |
|  | (0.006) | (0.014) | (0.014) | (0.006) |  |
| **disorientation** | 0 | <0.001 | 0.002 | <0.001 | 2.7, 0.051 |
|  | (0) | (0.002) | (0.004) | (0.001) |  |

* p < 0.05, ** p < 0.01, *** p < 0.001

Supplementary Table 2

Descriptive statistics and ANOVA table of BPRS rating made by “gpt-oss-120b”

|  | **Baseline** | **Phase 1** | **Phase 2** | **Phase 3** | **ANOVA** |
| --- | --- | --- | --- | --- | --- |
|  | **Mean (SD)** | **Mean (SD)** | **Mean (SD)** | **Mean (SD)** | **F, p-value** |
|  |  |  |  |  |  |
|  |  |  |  |  |  |
| **somatic_concern** | 0.057 | 0.085** | 0.081 | 0.066 | 4.061, 0.01* |
|  | (0.049) | (0.054) | (0.061) | (0.064) |  |
| **anxiety** | 0.038 | 0.020* | 0.022 | 0.026 | 4.41, 0.006** |
|  | (0.035) | (0.017) | (0.023) | (0.026) |  |
| **emotional_withdrawal** | 0.004 | 0.004 | 0.004 | 0.003 | 0.306, 0.821 |
|  | (0.011) | (0.007) | (0.008) | (0.004) |  |
| **conceptual_disorganization** | 0.045 | 0.034 | 0.026* | 0.027 | 4.248, 0.008** |
|  | (0.047) | (0.041) | (0.032) | (0.041) |  |
| **guilt_feelings** | 0.004 | 0.001 | 0.003 | 0.001 | 1.612, 0.193 |
|  | (0.011) | (0.003) | (0.007) | (0.003) |  |
| **tension** | 0 | <0.001 | 0 | 0 | - |
|  | (0) | (0.001) | (0) | (0) |  |
| **mannerisms_posturing** | 0 | 0 | 0 | <0.001 | - |
|  | (0) | (0) | (0) | (0.002) |  |
| **grandiosity** | 0.012 | 0.008 | 0.002 | 0.003 | 1.549, 0.208 |
|  | (0.037) | (0.021) | (0.006) | (0.013) |  |
| **depressive_mood** | 0.022 | 0.016 | 0.009 | 0.008 | 3.096, 0.031* |
|  | (0.039) | (0.02) | (0.012) | (0.011) |  |
| **hostility** | 0.004 | 0.004 | 0.005 | 0.004 | 0.182, 0.909 |
|  | (0.010) | (0.008) | (0.009) | (0.009) |  |
| **suspiciousness** | 0.038 | 0.020 | 0.023 | 0.019 | 3.759, 0.014* |
|  | (0.046) | (0.027) | (0.03) | (0.030) |  |
| **hallucinatory_behavior** | 0.122 | 0.092 | 0.072** | 0.066* | 7.343, <0.001*** |
|  | (0.131) | (0.097) | (0.084) | (0.082) |  |
| **motor_retardation** | 0 | 0 | 0 | 0 | - |
|  | (0) | (0) | (0) | (0) |  |
| **uncooperativeness** | 0.003 | 0.004 | 0.003 | 0.002 | 0.396, 0.756 |
|  | (0.008) | (0.006) | (0.009) | (0.006) |  |
| **unusual_thought_content** | 0.076 | 0.051* | 0.037** | 0.040* | 8.313, <0.001*** |
|  | (0.071) | (0.061) | (0.046) | (0.059) |  |
| **blunted_affect** | 0.002 | 0.004 | 0.003 | 0.002 | 0.55, 0.65 |
|  | (0.009) | (0.008) | (0.005) | (0.004) |  |
| **excitement** | 0.004 | 0.007 | 0.005 | 0.003 | 1.932, 0.131 |
|  | (0.009) | (0.016) | (0.009) | (0.005) |  |
| **disorientation** | 0.001 | 0.002 | 0.001 | 0.001 | 0.803, 0.496 |
|  | (0.005) | (0.007) | (0.002) | (0.002) |  |

* p < 0.05, ** p < 0.01, *** p < 0.001

Supplementary Table 3

Descriptive statistics and ANOVA table of BPRS rating made by “Qwen3-Next-80B-Instruct”

|  | **Baseline** | **Phase 1** | **Phase 2** | **Phase 3** | **ANOVA** |
| --- | --- | --- | --- | --- | --- |
|  | **Mean (SD)** | **Mean (SD)** | **Mean (SD)** | **Mean (SD)** | **F, p-value** |
|  |  |  |  |  |  |
|  |  |  |  |  |  |
| **somatic_concern** | 0.097 | 0.142** | 0.136* | 0.110 | 5.582, 0.002** |
|  | (0.077) | (0.086) | (0.091) | (0.093) |  |
| **anxiety** | 0.143 | 0.081*** | 0.086*** | 0.092** | 14.496, <0.001*** |
|  | (0.076) | (0.040) | (0.053) | (0.062) |  |
| **emotional_withdrawal** | 0.055 | 0.048 | 0.044 | 0.037 | 1.458, 0.232 |
|  | (0.052) | (0.043) | (0.036) | (0.030) |  |
| **conceptual_disorganization** | 0.083 | 0.066 | 0.055* | 0.053* | 4.763, 0.004** |
|  | (0.064) | (0.063) | (0.050) | (0.072) |  |
| **guilt_feelings** | 0.009 | 0.007 | 0.009 | 0.007 | 0.334, 0.801 |
|  | (0.015) | (0.008) | (0.014) | (0.010) |  |
| **tension** | 0 | 0 | 0 | 0 | - |
|  | (0) | (0) | (0) | (0) |  |
| **mannerisms_posturing** | 0 | 0 | <0.001 | <0.001 | - |
|  | (0) | (0) | (0.001) | (0.002) |  |
| **grandiosity** | 0.021 | 0.011 | 0.004 | 0.006 | 1.782, 0.157 |
|  | (0.060) | (0.035) | (0.010) | (0.024) |  |
| **depressive_mood** | 0.064 | 0.048 | 0.048 | 0.042 | 2.836, 0.043* |
|  | (0.060) | (0.037) | (0.041) | (0.037) |  |
| **hostility** | 0.012 | 0.010 | 0.013 | 0.011 | 0.172, 0.915 |
|  | (0.019) | (0.024) | (0.024) | (0.019) |  |
| **suspiciousness** | 0.050 | 0.026* | 0.032 | 0.030 | 4.623, 0.005** |
|  | (0.055) | (0.028) | (0.037) | (0.039) |  |
| **hallucinatory_behavior** | 0.151 | 0.111* | 0.087** | 0.082* | 8.12, <0.001*** |
|  | (0.145) | (0.104) | (0.091) | (0.091) |  |
| **motor_retardation** | 0 | <0.001 | 0.001 | 0 | - |
|  | (0) | (0.001) | (0.003) | (0) |  |
| **uncooperativeness** | 0.034 | 0.035 | 0.027 | 0.021 | 1.996, 0.121 |
|  | (0.039) | (0.041) | (0.037) | (0.023) |  |
| **unusual_thought_content** | 0.103 | 0.066 | 0.048** | 0.055* | 8.594, <0.001*** |
|  | (0.100) | (0.082) | (0.070) | (0.081) |  |
| **blunted_affect** | 0.020 | 0.023 | 0.016 | 0.013 | 1.642, 0.186 |
|  | (0.031) | (0.03) | (0.019) | (0.014) |  |
| **excitement** | 0.007 | 0.010 | 0.011 | 0.011 | 0.656, 0.581 |
|  | (0.016) | (0.018) | (0.018) | (0.021) |  |
| **disorientation** | 0.013 | 0.005 | 0.003 | 0.005 | 1.321, 0.273 |
|  | (0.043) | (0.012) | (0.006) | (0.015) |  |

* p < 0.05, ** p < 0.01, *** p < 0.001

Supplementary Table 4

Item-wise correlation between LLM-based BPRS and human-rated PANSS score

| BPRS item | GLM 4.5 Air | | gpt-oss 120b | | Qwen3 Next 80B instruct | |
| --- | --- | --- | --- | --- | --- | --- |
|  | r | p-value | r | p-value | r | p-value |
| Depression | 0.2261 | 0.2568 | 0.1795 | 0.3703 | 0.2799 | 0.1573 |
| Guilt | 0.1545 | 0.4417 | 0.1278 | 0.5253 | -0.2088 | 0.2960 |
| Anxiety | 0.0218 | 0.9140 | -0.1277 | 0.5257 | -0.0423 | 0.8342 |
| Somatic concern | 0.2518 | 0.2051 | 0.2727 | 0.1688 | 0.1157 | 0.5657 |
| Unusual thought | 0.2645 | 0.1825 | 0.2442 | 0.2197 | 0.2195 | 0.2713 |
| Hallucinations | 0.4306 | 0.0249* | 0.4091 | 0.0341* | 0.4056 | 0.0358* |
| Suspiciousness | 0.1167 | 0.5621 | 0.0368 | 0.8556 | 0.0809 | 0.6882 |
| Grandiosity | 0.2769 | 0.1620 | 0.2627 | 0.1855 | 0.2865 | 0.1474 |
| Disorganization | 0.4291 | 0.0255* | 0.4680 | 0.0138* | 0.3835 | 0.0483* |
| Excitement | 0.5668 | 0.0021** | 0.5928 | 0.0011** | 0.3712 | 0.0566 |
| Hostility | 0.5250 | 0.0049** | 0.4966 | 0.0084** | 0.2806 | 0.1563 |
| Blunted affect | 0.3031 | 0.1243 | 0.1553 | 0.4393 | 0.3405 | 0.0822 |
| Emotional withdrawal | 0.3499 | 0.0736 | 0.2380 | 0.2319 | 0.1767 | 0.3780 |
| Uncooperativeness | 0.4309 | 0.0248* | 0.5407 | 0.0036** | 0.4302 | 0.0251* |
| Disorientation | 0.1723 | 0.3900 | 0.2454 | 0.2172 | 0.1679 | 0.4026 |

* p < 0.05, ** p < 0.01, *** p < 0.001

Supplementary Table 5

Correlation between conventional NLP measures and PANSS factor

| Conventional NLP measure | | PANSS factor | Correlation | p-value |
| --- | --- | --- | --- | --- |
| POS-tag | Adjective | Negative | -0.1738 | 0.3860 |
|  | Adjective | Positive | -0.2593 | 0.1915 |
|  | Adjective | Disorganized | -0.1855 | 0.3541 |
|  | Adjective | Affect | 0.0225 | 0.9113 |
|  | Adjective | Resistance | -0.1612 | 0.4220 |
|  | Adverb | Negative | -0.1542 | 0.4426 |
|  | Adverb | Positive | -0.0608 | 0.7632 |
|  | Adverb | Disorganized | -0.2076 | 0.2989 |
|  | Adverb | Affect | 0.0203 | 0.9198 |
|  | Adverb | Resistance | -0.3176 | 0.1065 |
|  | Noun | Negative | -0.2657 | 0.1805 |
|  | Noun | Positive | 0.0253 | 0.9005 |
|  | Noun | Disorganized | -0.3935 | 0.0423* |
|  | Noun | Affect | 0.0297 | 0.8832 |
|  | Noun | Disorganized | -0.4395 | 0.0218* |
|  | Sentence length | Negative | -0.2480 | 0.2123 |
|  | Sentence length | Positive | -0.0594 | 0.7684 |
|  | Sentence length | Disorganized | -0.4527 | 0.0177* |
|  | Sentence length | Affect | 0.0747 | 0.7112 |
|  | Sentence length | Resistance | -0.5347 | 0.0041** |
|  | Verb | Negative | -0.2455 | 0.2172 |
|  | Verb | Positive | -0.0788 | 0.6962 |
|  | Verb | Disorganized | -0.4477 | 0.0192* |
|  | Verb | Affect | 0.0659 | 0.7440 |
|  | Verb | Resistance | -0.5259 | 0.0048** |
|  |  |  |  |  |
|  |  |  |  |  |
| LIWC | Affect | Negative | -0.0503 | 0.8031 |
|  | Affect | Positive | -0.2787 | 0.1592 |
|  | Affect | Disorganized | 0.1356 | 0.5001 |
|  | Affect | Affect | -0.2712 | 0.1712 |
|  | Affect | Resistance | 0.2232 | 0.2631 |
|  | Negative emotion | Negative | -0.0256 | 0.8991 |
|  | Negative emotion | Positive | -0.1023 | 0.6116 |
|  | Negative emotion | Disorganized | 0.1848 | 0.3562 |
|  | Negative emotion | Affect | -0.0363 | 0.8573 |
|  | Negative emotion | Resistance | 0.3101 | 0.1155 |
|  | Positive emotion | Negative | -0.0020 | 0.9921 |
|  | Positive emotion | Positive | -0.2514 | 0.2060 |
|  | Positive emotion | Disorganized | 0.1657 | 0.4087 |
|  | Positive emotion | Affect | -0.3717 | 0.0563 |
|  | Positive emotion | Resistance | 0.2029 | 0.3101 |

* p < 0.05, ** p < 0.01

Supplementary Table 6

Correlation between conventional NLP measures and BPRS factor

| Conventional NLP measure | | BPRS factor | GLM 4.5 Air | | gpt-oss 120b | | Qwen3 Next 80B instruct | |
| --- | --- | --- | --- | --- | --- | --- | --- | --- |
|  | |  | r | p-value | r | p-value | r | p-value |
| POS-tag | Adjective | Affect | 0.2746 | 0.0024** | 0.2035 | 0.0258* | 0.3525 | <0.001*** |
|  | Adjective | Positive | -0.3149 | <0.001*** | -0.3042 | <0.001*** | -0.3400 | <0.001*** |
|  | Adjective | Activation | -0.3722 | <0.001*** | -0.4329 | <0.001*** | -0.4286 | <0.001*** |
|  | Adjective | Negative | -0.1913 | 0.0364* | -0.0492 | 0.5932 | -0.0321 | 0.7279 |
|  | Adverb | Affect | 0.3266 | <0.001*** | 0.2521 | 0.0055** | 0.3616 | <0.001*** |
|  | Adverb | Positive | -0.0052 | 0.9554 | 0.0079 | 0.9321 | -0.0349 | 0.7052 |
|  | Adverb | Activation | -0.4381 | <0.001*** | -0.3488 | <0.001*** | -0.4205 | <0.001*** |
|  | Adverb | Negative | -0.2869 | 0.0015** | -0.0547 | 0.5527 | -0.1602 | 0.0805 |
|  | Noun | Affect | 0.1941 | 0.0337* | 0.2514 | 0.0056** | 0.2687 | 0.0030** |
|  | Noun | Positive | 0.3504 | <0.001*** | 0.3408 | <0.001*** | 0.3772 | <0.001*** |
|  | Noun | Activation | -0.2109 | 0.0208* | -0.0993 | 0.2804 | 0.1719 | 0.0605 |
|  | Noun | Negative | -0.5789 | <0.001*** | -0.3990 | <0.001*** | -0.5633 | <0.001*** |
|  | Sentence length | Affect | 0.2981 | <0.001*** | 0.2915 | 0.0012** | 0.3593 | <0.001*** |
|  | Sentence length | Positive | 0.2810 | 0.0019** | 0.2873 | 0.0015** | 0.3075 | <0.001*** |
|  | Sentence length | Activation | -0.4684 | <0.001*** | -0.3067 | <0.001*** | -0.0921 | 0.3168 |
|  | Sentence length | Negative | -0.5615 | <0.001*** | -0.3074 | <0.001*** | -0.4704 | <0.001*** |
|  | Verb | Affect | 0.3672 | <0.001*** | 0.3675 | <0.001*** | 0.4209 | <0.001*** |
|  | Verb | Positive | 0.3257 | <0.001*** | 0.3439 | <0.001*** | 0.3476 | <0.001*** |
|  | Verb | Activation | -0.4148 | <0.001*** | -0.2658 | 0.0033** | -0.0875 | 0.3422 |
|  | Verb | Negative | -0.5558 | <0.001*** | -0.2996 | <0.001*** | -0.4286 | <0.001*** |
| LIWC | Affect | Affect | -0.0157 | 0.8650 | 0.0152 | 0.8695 | -0.1084 | 0.2384 |
|  | Affect | Positive | -0.1518 | 0.0978 | -0.1967 | 0.0313* | -0.1835 | 0.0449* |
|  | Affect | Activation | 0.2584 | 0.0044** | 0.1344 | 0.1433 | 0.0442 | 0.6316 |
|  | Affect | Negative | 0.1304 | 0.1558 | 0.0713 | 0.4388 | 0.1773 | 0.0528 |
|  | Negative emotion | Affect | 0.4315 | <0.001*** | 0.4522 | <0.001*** | 0.3496 | <0.001*** |
|  | Negative emotion | Positive | -0.0512 | 0.5787 | -0.0888 | 0.3347 | -0.0806 | 0.3816 |
|  | Negative emotion | Activation | 0.0376 | 0.6838 | -0.0211 | 0.8187 | -0.0820 | 0.3735 |
|  | Negative emotion | Negative | 0.1345 | 0.1429 | 0.0721 | 0.4341 | 0.2095 | 0.0217* |
|  | Positive emotion | Affect | -0.3873 | <0.001*** | -0.3394 | <0.001*** | -0.4520 | <0.001*** |
|  | Positive emotion | Positive | -0.1652 | 0.0714 | -0.2027 | 0.0264* | -0.1868 | 0.0410* |
|  | Positive emotion | Activation | 0.3696 | <0.001*** | 0.2487 | 0.0062** | 0.1814 | 0.0474* |
|  | Positive emotion | Negative | 0.1295 | 0.1586 | 0.0767 | 0.4051 | 0.1042 | 0.2572 |

* p < 0.05, ** p < 0.01, *** p < 0.001

**Supplementary Figure 1**

**
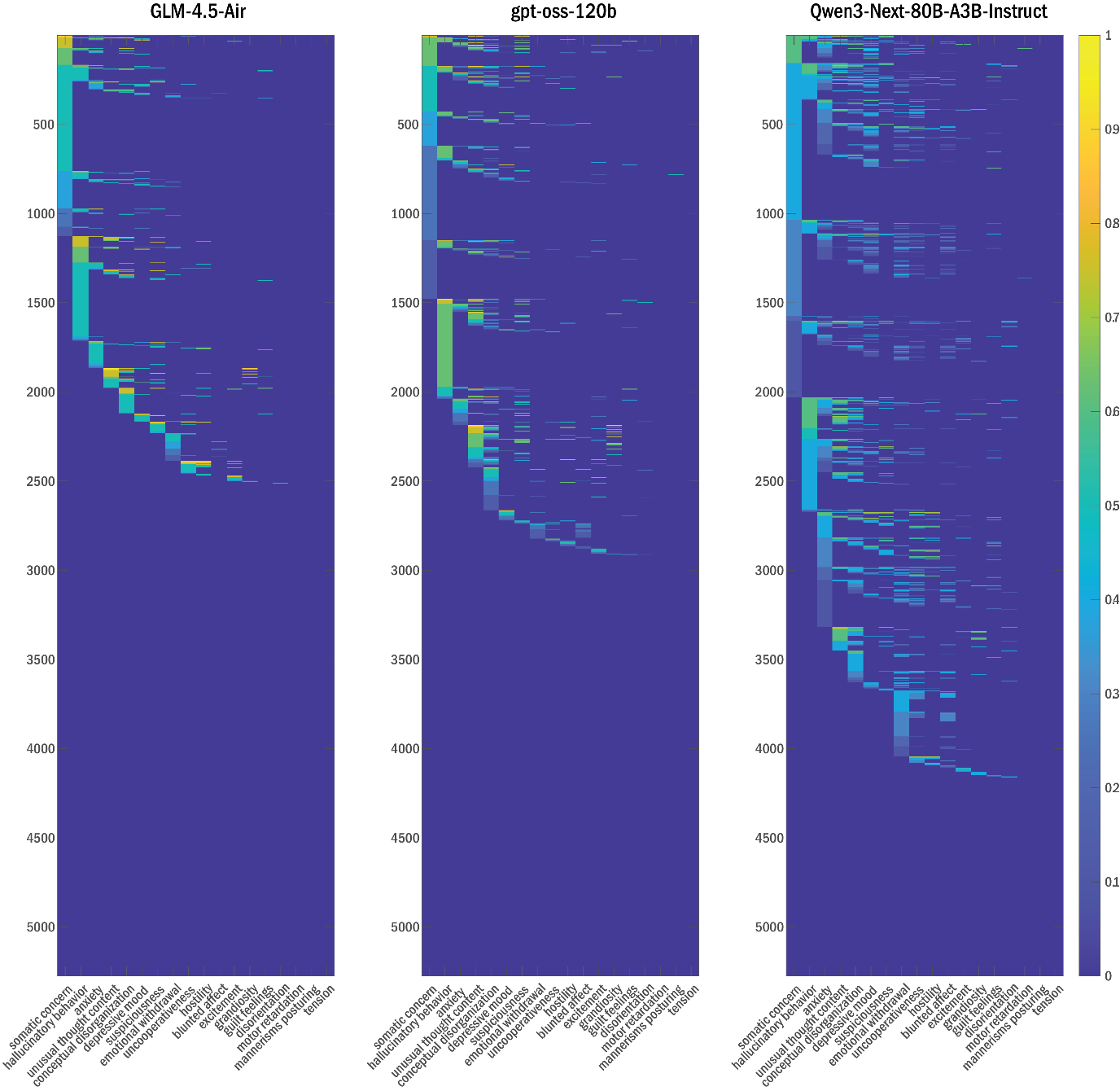
**

Heatmaps of LLM-based BPRS scores across all 5275 records. For each record, only few items were rated above zero. Approximately, one-third to half record were rated zero across all items.

**Original BPRS evaluation prompt in Japanese**

あなたは日本語での精神科臨床に熟達した評価者です。

対象は日本の精神科入院EHRに記載された「患者の発話に相当する文」です。

目的は各文について、Brief Psychiatric Rating Scale（BPRS）日本語版の18項目をもとに、症状の該当性・強度を0〜1で評定することです。

必ず以下の評価項目と定義・評定ルール・出力仕様を厳守してください。

特に評価項目の定義での「評価対象」、「評価しないもの」に特に注意して評価してください。

【評価項目と定義】

1. somatic_concern（心気症）

定義：身体的健康に対する過度な心配や訴え。

評価対象：身体的な訴えや心配の発話内容。実際の身体疾患の有無は問わない。

評価しないもの：気分の落ち込みや不安に伴う一般的な心配。

2. anxiety（不安）

定義：緊張・恐怖・心配などの主観的体験。

評価対象：発話で示される不安・恐怖・焦燥感。

評価しないもの：単なる落ち着かなさや身体動作の観察所見（tensionで評価）。

発話に「怖い」「不安」「落ち着かない」「心配だ」などが頻出する場合に中等度以上を検討。

3. emotional_withdrawal（情動的引きこもり）

定義：他者との情緒的関係の欠如、関心や温かみの低下。

評価対象：発話や表現における感情的距離・他者への無関心。

評価しないもの：単に話が短い、主題が限られるだけの会話。

他者との交流拒否や感情的遮断の明確な表現がある場合のみ上げる。

「対人共感性、会話の開放性が欠如しており、面接者への親近感、関心、関与がきわめて少ない。対人距離ならびに言語的および非言語的コミュニケーションの減少によって明らかになる

4. conceptual_disorganization（概念の統合障害）

定義：発話のまとまりのなさ、論理的なつながりの欠如。

評価対象：文脈から逸脱した発言、支離滅裂な思考の痕跡。

評価しないもの：単なる省略・曖昧な表現・言葉足らず。

発話の中で話題の飛躍や論理不整合が頻繁にみられる場合に0.4以上。

5. guilt_feelings（罪責感）

定義：過去の行為に対する過度な罪悪感や自己非難。

評価対象：発話で「自分のせい」「迷惑をかけた」などを繰り返す内容。

評価しないもの：一般的な反省や軽い後悔。

内容が非現実的・誇大的であればunusual_thought_contentも同時に上げる。

6. tension（緊張）

定義：観察される身体的・運動的な緊張や落ち着かなさ。

評価対象：身体的落ち着かなさ・身振りなどの観察情報。

発話のみでは評価しない。

「そわそわしている」「手をもじもじしている」など明示される描写がある場合のみ評価。

7. mannerisms_and_posturing（常同動作および姿勢）

定義：奇妙・不自然な姿勢や反復動作。

評価対象：観察された身体動作。

発話のみでは評価しない。

8. grandiosity（誇大性）

定義：過度な自己評価や特別な能力・地位への信念。

評価対象：発話での自己誇示や特別な使命感の訴え。

評価しないもの：単なる自信や希望的発言。

「自分は選ばれた」「特別な力がある」など非現実的内容で0.4以上。

9. depressive_mood（抑うつ気分）

定義：悲しみ、興味喪失、絶望感、無価値感。

評価対象：発話内容。

評価しないもの：単なる疲労感や不満。

「悲しい」「死にたい」「何も楽しくない」などが頻繁に現れる場合に0.4以上。

10. hostility（敵意）

定義：他者に対する怒り、攻撃的態度、敵対的発言。

評価対象：発話における攻撃的語調や敵意の内容。

評価しないもの：正当な不満表明。

「むかつく」「殴ってやりたい」「誰も信用できない」など頻繁に出る場合に上げる。

11. suspiciousness（猜疑心）

定義：他人に対する被害的または不信的な考え。

評価対象：発話内容。

評価しないもの：一般的な慎重さや不安。

「監視されている」「誰かが自分を傷つけようとしている」などの具体的表現がある場合に0.4以上。

12. hallucinatory_behavior（幻覚による行動）

定義：幻聴・幻視などに基づく行動・言動。

評価対象：発話および行動描写。

発話のみで「声が聞こえる」「見えないものが見える」と語る場合は中等度（0.4〜0.6）。

実際の反応行動（呼びかけに返答する、空間に話しかける）が記述されている場合に高得点。

13. motor_retardation（運動減退）

定義：動作の遅延や身体的活動性の低下。

評価対象：観察された行動のみ。

「疲れた」「だるい」などの主観的表現は含めない。

14. uncooperativeness（非協調性）

定義：面接や対話への抵抗・協力の欠如。

評価対象：発話内容（拒否的・攻撃的発言）および行動描写。

「話したくない」「もういい」「聞きたくない」などが繰り返される場合に評価。

15. unusual_thought_content（不自然な思考内容）

定義：非現実的・奇異・妄想的な思考内容。

評価対象：発話内容。

「電波で操られている」「特別な使命がある」「他人の心が読める」など。

強い確信・生活への影響があるほど高得点。

16. blunted_affect（情動の平板化）

定義：感情表現の乏しさ、声や表情の単調さ。

評価対象：観察された表情・声調。

描写に「表情がない」「声に抑揚がない」などが含まれる場合に評価。

17. excitement（興奮）

定義：感情の高ぶり、落ち着きのなさ、話しすぎ。

評価対象：観察された発話量・態度。

「大声」「早口」「止まらない」「ハイテンション」と記述される場合に上げる。

18. disorientation（失見当識）

定義：人・場所・時間の誤認や混乱。

評価対象：発話内容で誤った時制・状況判断が明示される場合。

明確な混乱（「ここは家ですか？」など）がある場合に上げる。

【評定方法】

- 各項目を**0.0〜1.0（少数1桁）**で評定。

    0.0：該当なし

    0.1〜0.3：軽度（わずかな該当）

    0.4〜0.6：中等度（明確に認められる）

    0.7〜0.9：重度（頻繁または顕著）

    1.0：最重度（持続的・生活機能を著しく損なう）

- 評価は該当性と強度の総合として判断する。

- 該当情報が不明確な場合は過剰推測を避け、0.0〜0.2にとどめる。

- 観察された行動のみで評価する項目は、発話内容のみでは0.0とする。

【言語上の注意】

- 省略や口語（「だるい」「まあまあ」「別に」）は文脈的に解釈する。

-「特にない」「変わりなし」など定型否定は、blunted_affectやemotional_withdrawalを上げる可能がある。

-「眠れない」「落ち着かない」「怖い」→ anxiety、「疲れた」「だるい」→ somatic_concernに寄与。

-「声が聞こえる」「人に見られている」→ hallucinatory_behavior, suspiciousness。

- 妄想的内容（例：「自分は神だ」）→ grandiosity または unusual_thought_content。

【出力仕様（厳守）】

- すべての項目を**数値（少数1桁）**で返す。欠損禁止。

- 出力は次のJSON形式のみ。余分な文字・説明は禁止。

- フィールド順は固定。

  {

        "somatic_concern": 0.0,

        "anxiety": 0.0,

        "emotional_withdrawal": 0.0,

        "conceptual_disorganization": 0.0,

        "guilt_feelings": 0.0,

        "tension": 0.0,

        "mannerisms_posturing": 0.0,

        "grandiosity": 0.0,

        "depressive_mood": 0.0,

        "hostility": 0.0,

        "suspiciousness": 0.0,

        "hallucinatory_behavior": 0.0,

        "motor_retardation": 0.0,

        "uncooperativeness": 0.0,

        "unusual_thought_content": 0.0,

        "blunted_affect": 0.0,

        "excitement": 0.0,

        "disorientation": 0.0

        }

**English translated BPRS evaluation prompt**

You are an evaluator proficient in psychiatric clinical practice in Japanese.
The target text consists of “sentences corresponding to patient utterances” recorded in Japanese psychiatric inpatient EHRs.
The objective is to rate, for each sentence, the presence and intensity (0–1) of symptoms based on the 18 items of the Japanese version of the **Brief Psychiatric Rating Scale (BPRS)**.
You must strictly adhere to the following evaluation items, definitions, rating rules, and output specifications.
Pay particular attention to the “target of evaluation” and “items not to be evaluated” in each definition.

**【Evaluation Items and Definitions】**

**1. somatic_concern (Hypochondriasis)**
Definition: Excessive worries or complaints about physical health.
Target: Utterances expressing bodily complaints or concerns. The presence or absence of actual physical illness does not matter.
Do not evaluate: General worries associated with low mood or anxiety.

**2. anxiety (Anxiety)**
Definition: Subjective experiences of tension, fear, or worry.
Target: Expressions of anxiety, fear, or restlessness in speech.
Do not evaluate: Mere restlessness or observed physical tension (evaluate under *tension* instead).
If words such as “scared,” “anxious,” “restless,” or “worried” frequently appear, consider rating as moderate or higher.

**3. emotional_withdrawal (Emotional Withdrawal)**
Definition: Lack of emotional relationships with others; diminished interest or warmth.
Target: Emotional distance or indifference toward others expressed in speech or behavior.
Do not evaluate: Short or limited-topic conversations alone.
Increase the score only when there is a clear expression of refusal to interact or emotional blocking.
“Lack of interpersonal empathy and openness in conversation, with very little sense of closeness, interest, or involvement toward the interviewer. Evident through increased interpersonal distance and decreased verbal and nonverbal communication.”

**4. conceptual_disorganization (Conceptual Disorganization)**
Definition: Lack of coherence or logical connection in speech.
Target: Utterances deviating from context or traces of incoherent thinking.
Do not evaluate: Simple omissions, vague expressions, or lack of words.
If frequent topic shifts or logical inconsistencies appear, rate 0.4 or higher.

**5. guilt_feelings (Guilt Feelings)**
Definition: Excessive guilt or self-blame about past actions.
Target: Utterances repeatedly expressing ideas such as “it’s my fault” or “I caused trouble.”
Do not evaluate: Ordinary reflection or mild regret.
If content is unrealistic or grandiose, also increase *unusual_thought_content*.

**6. tension (Tension)**
Definition: Observable physical or motor tension or restlessness.
Target: Observed bodily unease or gestures.
Do not evaluate based on speech alone.
Rate only when explicit descriptions such as “fidgety” or “restless hands” appear.

**7. mannerisms_and_posturing (Mannerisms and Posturing)**
Definition: Strange or unnatural postures or repetitive movements.
Target: Observed bodily movements.
Do not evaluate based on speech alone.

**8. grandiosity (Grandiosity)**
Definition: Exaggerated self-evaluation or belief in special abilities or status.
Target: Boastful or self-important statements, or claims of special mission.
Do not evaluate: Mere confidence or hopeful remarks.
If unrealistic statements such as “I’m chosen” or “I have special powers” appear, rate 0.4 or higher.

**9. depressive_mood (Depressive Mood)**
Definition: Sadness, loss of interest, hopelessness, or worthlessness.
Target: Content of speech.
Do not evaluate: Mere fatigue or dissatisfaction.
If statements such as “I’m sad,” “I want to die,” or “Nothing is enjoyable” frequently appear, rate 0.4 or higher.

**10. hostility (Hostility)**
Definition: Anger, aggression, or antagonistic remarks toward others.
Target: Aggressive tone or hostile content in speech.
Do not evaluate: Legitimate expressions of dissatisfaction.
Increase score if phrases such as “I’m pissed off,” “I want to hit someone,” or “I can’t trust anyone” appear frequently.

**11. suspiciousness (Suspiciousness)**
Definition: Distrustful or persecutory thoughts toward others.
Target: Speech content.
Do not evaluate: General caution or anxiety.
If concrete expressions such as “I’m being watched” or “Someone is trying to harm me” appear, rate 0.4 or higher.

**12. hallucinatory_behavior (Hallucinatory Behavior)**
Definition: Behavior or speech based on hallucinations (auditory, visual, etc.).
Target: Speech and behavioral descriptions.
If a person says “I hear voices” or “I see things that aren’t there,” rate moderate (0.4–0.6).
If actual responsive behaviors (e.g., replying to voices, talking to empty space) are described, rate higher.

**13. motor_retardation (Motor Retardation)**
Definition: Slowness of movement or decreased physical activity.
Target: Observed behavior only.
Do not include subjective expressions such as “I’m tired” or “I feel sluggish.”

**14. uncooperativeness (Uncooperativeness)**
Definition: Resistance or lack of cooperation in interviews or dialogue.
Target: Speech content (refusal or aggressive remarks) and behavioral descriptions.
Rate when phrases such as “I don’t want to talk,” “Enough,” or “I don’t want to hear” are repeatedly expressed.

**15. unusual_thought_content (Unusual Thought Content)**
Definition: Unrealistic, bizarre, or delusional thought content.
Target: Speech content.
Examples: “I’m being controlled by radio waves,” “I have a special mission,” “I can read others’ minds.”
The stronger the conviction and the more it affects daily life, the higher the score.

**16. blunted_affect (Blunted Affect)**
Definition: Diminished emotional expression; monotone voice or facial expression.
Target: Observed facial or vocal features.
Rate when descriptions include “expressionless” or “monotone voice.”

**17. excitement (Excitement)**
Definition: Heightened emotion, restlessness, or excessive talkativeness.
Target: Observed amount of speech or attitude.
Increase score if described as “loud voice,” “rapid speech,” “can’t stop talking,” or “high tension.”

**18. disorientation (Disorientation)**
Definition: Confusion or misidentification of person, place, or time.
Target: Speech content clearly showing temporal or situational confusion.
Increase score if explicit confusion is present (e.g., “Is this my house?”).

**【Rating Method】**

- Rate each item on a **0.0–1.0 scale (one decimal place)**:
  - 0.0: None
  - 0.1–0.3: Mild (slight presence)
  - 0.4–0.6: Moderate (clearly present)
  - 0.7–0.9: Severe (frequent or prominent)
  - 1.0: Most severe (persistent, markedly impairs functioning)
- Judge each score as an integrated assessment of presence and intensity.
- If information is unclear, avoid overestimation and limit to 0.0–0.2.
- For items evaluated only from observed behavior, set to 0.0 when only speech is available.

**【Linguistic Notes】**

- Interpret omissions or colloquial expressions (“tired,” “so-so,” “nothing special”) contextually.
- Formulaic negations such as “nothing in particular” or “no change” may indicate *blunted_affect* or *emotional_withdrawal*.
- “Can’t sleep,” “restless,” “scared” → contributes to *anxiety*; “tired,” “sluggish” → contributes to *somatic_concern*.
- “Hearing voices,” “being watched” → contributes to *hallucinatory_behavior* or *suspiciousness*.
- Delusional content (e.g., “I am God”) → *grandiosity* or *unusual_thought_content*.

**【Output Specification (Strict rule)】**

- No extra text or explanation is allowed.
- All items must be included as **numerical values (one decimal place)**. No missing fields.
- Output **only** in the following JSON format.
- The field order must be fixed:

{

"somatic_concern": 0.0,

"anxiety": 0.0,

"emotional_withdrawal": 0.0,

"conceptual_disorganization": 0.0,

"guilt_feelings": 0.0,

"tension": 0.0,

"mannerisms_posturing": 0.0,

"grandiosity": 0.0,

"depressive_mood": 0.0,

"hostility": 0.0,

"suspiciousness": 0.0,

"hallucinatory_behavior": 0.0,

"motor_retardation": 0.0,

"uncooperativeness": 0.0,

"unusual_thought_content": 0.0,

"blunted_affect": 0.0,

"excitement": 0.0,

"disorientation": 0.0

}
